# Multicenter cohort study of the effect of Crinone® progesterone vaginal gel alone to prepare the endometrium for frozen–thawed blastocyst transfer in a hormone replacement cycle

**DOI:** 10.1111/jog.16325

**Published:** 2025-06-02

**Authors:** Akiko Takashima, Yukihiro Shibui, Toshio Hamatani, Akihiko Suenaga, Yoshimitsu Kuwabara, Fuminori Kimura, Koji Nakagawa, Yusuke Fukuda, Shoji Tokunaga, Yukiko Katagiri

**Affiliations:** ^1^ Department of Obstetrics and Gynecology Toho University Medical Center Sakura Hospital Chiba Japan; ^2^ Cinema Art Clinic Tokyo Japan; ^3^ Department of Obstetrics and Gynecology Keio University School of Medicine Tokyo Japan; ^4^ Nishitan ART clinic Shinagawa Tokyo Japan; ^5^ Department of Obstetrics and Gynecology Nippon Medical School Tokyo Japan; ^6^ Department of Obstetrics and Gynecology Shiga University of Medical Science Shiga Japan; ^7^ Division of Reproductive Medicine Sugiyama Clinic Tokyo Japan; ^8^ Department of Obstetrics and Gynecology Faculty of Medicine, Toho University Tokyo Japan; ^9^ The Medical Information Center, Kyushu University Hospital Fukuoka Japan

**Keywords:** frozen–thawed embryo transfer, hormone replacement, luteal phase support, pregnancy rate, progesterone

## Abstract

**Aim:**

Progesterone has to be administered to prepare the endometrium for frozen–thawed blastocyst transfer (FBT) in a hormone replacement cycle (HRC). The objective of this study was to investigate the efficacy and safety of using Crinone® progesterone gel alone in HRC‐FBT.

**Methods:**

In this multicenter prospective study, HRC‐FBT was performed with blastocysts with a Gardner's classification of 3BB or better, and application of 90 mg/day of vaginal gel (Crinone, Merck BioPharma, Tokyo) was started when the endometrial thickness reached 8 mm or more. The primary endpoint was the clinical pregnancy rate (CPR). Safety endpoints included genital bleeding and other adverse events. Recruitment started in May 2018.

**Results:**

A total of 181 patients were enrolled, and 156 were included in the efficacy analysis. The overall CPR was 41.7% (65/156). In patients younger than 38 years (*n* = 113), the CPR was 48.7% (55/113), and in those aged 38 years or older (*n* = 43), it was 23.3% (10/43). The overall CPR was comparable to that observed in the Japan Society of Obstetrics and Gynecology ART2020 National Survey, which reported a pregnancy rate of 36.3% in frozen embryo transfer cycles. Adverse events such as light genital bleeding occurred before and after pregnancy in some patients, but at a low frequency (<10%).

**Conclusions:**

Use of Crinone progesterone vaginal gel alone is adequate in HRC‐FBT and is not associated with any safety issues.

## INTRODUCTION

In frozen–thawed embryo transfer (FET), two methods are available for preparing the endometrium: natural ovulatory cycle (NOC) and hormone replacement cycle (HRC). Progesterone is needed to maintain pregnancy and is primarily secreted by the corpus luteum from the start of the implantation period in the early stages of pregnancy; however, in HRC, ovulation and subsequent corpus luteum development do not occur because relatively high concentrations of estradiol are administered even in the initial phase. Consequently, no endogenous progesterone is secreted, and progesterone is administered to achieve and maintain pregnancy.

In Japan, vaginal progesterone gel (OneCrinone® vaginal gel 90 mg, Merck Biopharma, Tokyo, Japan; hereinafter referred to as Crinone) has been available since 2016 for use as an endometrial preparation for FET in both HRC and NOC. The phase III study of Crinone demonstrated its efficacy and safety in patients undergoing in vitro fertilization and embryo transfer (IVF‐ET) with fresh embryos in Japan.^1^ However, recently, 62% of assisted reproductive technology (ART) embryo transfer treatments in Japan have been performed by FET, and this number is expected to increase annually.^2^ Furthermore, 92.7% of babies born by ART treatment are conceived by frozen embryos. According to the Japan Society of Obstetrics and Gynecology (JSOG) ART2020 National Survey, the pregnancy rate per number of embryo transfer cycles was 36.3% for FET but only 21.2% for fresh embryo transfer.^3^ Consequently, FET has become the preferred ART treatment in Japan.

Some authors have expressed concern that HRC‐FET requires higher doses of exogenous progesterone supplementation than FET in a NOC.,^4^, ^5^, ^6^ However, no prospective data are available on the effect of progesterone supplementation with Crinone 90 mg alone in HRC‐FET. Therefore, this study aimed to evaluate the efficacy and safety of daily use of Crinone 90 mg alone in HRC‐FET in Japan. The JSOG prohibits egg donations in Japan, but the mean age of patients receiving ART treatment in Japan is close to 40 years (37.8 ± 4.8 years),^2^ so the results of this study will be useful for women of this age group who receive FET.

## MATERIALS AND METHODS

### Study design

This study (J‐FOCAL) was designed as a multicenter single‐arm phase IV trial to evaluate whether Crinone as the sole agent for progesterone supplementation safely improves the clinical pregnancy rate (CPR) in HRC‐FET. There are two stages of embryo transfer: cleavage‐stage embryo transfer and blastocyst transfer; this study targeted frozen–thawed blastocyst transfer (FBT) among frozen–thawed embryo transfers.

The study protocol was approved by the central review board of the Clinical Research Network Fukuoka (ethical review number 18‐C13). The study was registered in the UMIN Clinical Trials Registry (registration number UMIN000031115) and was performed in accordance with the Declaration of Helsinki. All procedures were performed in accordance with the ethical standards of the relevant committees on human experimentation (institutional and national) and with the Helsinki Declaration of 1964 and its later amendments.

### Sample size calculation

To estimate the sample size, we calculated the number of cases for which the width of the confidence interval of the proportion was expected to be smaller than a predefined value (see below) and determined the sample size required to ensure that the requisite number of cases could be obtained. According to data from previous ART clinical trials^7^, ^8^ and observational studies,^9^ the CPR per number of embryo transfers was assumed to range from 30% to 40%, so we calculated the width of the confidence interval for the rate within this range. On the basis of the results and in view of the capacity of case pooling, the precision of the estimated CPR for calculating the required number of cases was set at less than 15% of the width of the 95% confidence interval. Therefore, recruiting at least 161 cases would enable the CPR to be estimated with a width of less than 15% of the 95% confidence interval. Assuming a dropout rate of approximately 10% and the identification of some ineligible cases after enrollment, the planned number of participants was set at 180.

### Study population

The study population comprised healthy, premenopausal Japanese women who planned to undergo FBT and were 42 years of age or younger on the day of blastocyst transfer; the age limit was chosen because the mean age of patients receiving ART treatment in Japan is nearly 40 years (37.8 ± 4.8 years).^2^ Patients were recruited at eight hospitals and clinics in Japan from May 2018 until a sufficient number of participants had been recruited.

All patients provided written informed consent to participate in the study and were enrolled after being screened for inclusion and exclusion criteria (see Appendix 1).

### Study treatments and interventions

For FBT, each study site could use its usual estrogen preparation for HRC. The types and methods of estrogen preparations were administered according to the protocols of each facility. Once the endometrium had reached sufficient thickness (≥8 mm), daily administration of Crinone was initiated; the estrogen preparation was continued until the day of the pregnancy test (see below). Patients were instructed to administer Crinone at approximately the same time every day (preferably in the morning). Crinone gel is provided in individual applicators and is administered by inserting the applicator into the vagina. In this study, the daily dosage of Crinone was fixed, the administration method and timing were implemented in accordance with the instructions in the package insert, and no modifications were permitted.

The initial day of Crinone administration was designated as day 0, and FBT was performed on day 4 or 5. In cases where a pregnancy had been established on a previous FET, the same protocol was used as in the former FET cycle.

A pregnancy test was performed by measuring the serum beta human chorionic gonadotropin (β‐hCG) concentration on day 19 (± 2 days). If the test result was negative, the administration of Crinone and the estrogen preparation was discontinued, but if the result was positive, the administration of both products was continued. In patients with a positive β‐hCG, the presence of a gestational sac in the uterine cavity was evaluated by transvaginal ultrasound (TVUS) at 5 to 6 weeks. A clinical pregnancy was defined as TVUS confirmation of a gestational sac inside or outside the uterine cavity at 5 to 6 weeks of gestation. In contrast, a biochemical pregnancy was defined as a TVUS examination at 5 to 6 weeks of gestation that showed the absence of a gestational sac and a confirmed miscarriage, accompanied by a positive serum β‐hCG on day 19 (± 2) (Table 1). The discontinuation and completion criteria of the protocol treatment are listed in Appendix 2.

**TABLE 1:** Study schedule.

Event	6 months before enrollment	Day −7 to −21	Informed consent and enrollment	Day 0^a^	Day 4–5	Day 19 (± 2 days)	Five to 6 weeks' gestation	Seven to 8 weeks' gestation
	Screening	Start of estrogen administration^c^	Explanation and IC; enrollment	Confirmation of endometrial thickness; start of Crinone administration^d^	Embryo transfer	Pregnancy test; serum β‐hCG or hCG quantification test	Confirmation of clinical pregnancy^f^	Confirmation of fetal heartbeat
Obtain IC			〇					
Patient characteristics: pregnancies and number of births, etc.	〇							
Weight, height	〇							
Cervical cytology^b^	〇							
HPV test^b^	〇							
Hormone tests: Serum FSH Serum TSH Serum PRL	〇							
Embryo transfer					〇			
Pregnancy test: serum β‐hCG or hCG						〇		
TVUS	〇			〇			〇	〇
Concomitant drugs	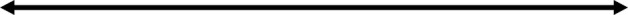							
Crinone^e^ administration status				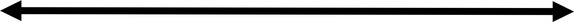				
Confirmation of safety				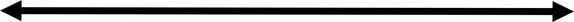				

Abbreviations: FSH, follicle‐stimulating hormone; hCG, human chorionic gonadotropin; HPV, human papillomavirus; IC, informed consent; PRL, prolactin; TSH, thyroid‐stimulating hormone; TVUS, transvaginal ultrasound.

^a^ The start date of administration of Crinone vaginal gel was defined as day 0.

^b^ Testing was not required if the procedure was performed within 12 months prior to the date of informed consent, the cervical cytology results were normal, and the human papillomavirus (HPV) test was negative. The patient was allowed to report the results of previous testing (to be recorded in the medical record). If test results were out of date or unknown, cervical cytology and, if necessary, HPV testing were to be performed.

^c^ For estradiol, conventional methods commonly practiced at each site were implemented.

^d^ Endometrial thickness of ≥8 mm was required to start administering Crinone vaginal gel.

^e^ Crinone vaginal gel was to be administered within the period according to the package insert. If the results of the day 19 pregnancy test were negative or if miscarriage or ectopic pregnancy was confirmed, administration was discontinued.

^f^ A clinical pregnancy was defined as a confirmed uterine or ectopic pregnancy on transvaginal ultrasound at 5 to 6 weeks' gestation.

**FIGURE 1:**
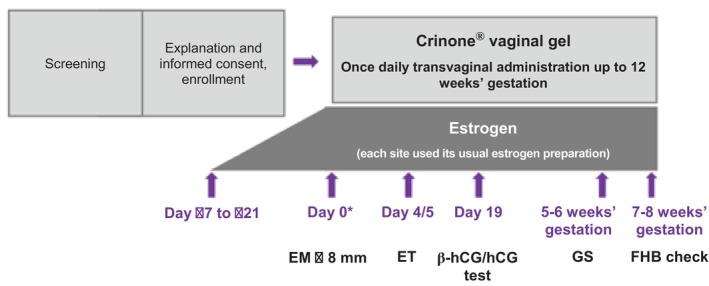
Study schema. EM, endometrium; ET, embryo transfer; FHB, fetal heart beat; GS, gestational sac; hCG, human chorionic gonadotropin. *The start of Crinone administration was set as day 0; Crinone was started if the endometrial thickness was at least 8 mm.

If a clinical pregnancy was confirmed, administration of Crinone was continued in accordance with the package insert, and the estrogen preparation was also continued. In the event of miscarriage in the first 5 to 6 weeks of gestation, Crinone and the estrogen preparation were discontinued as soon as the miscarriage was confirmed. Fetal heartbeat was confirmed by TVUS at 7 to 8 weeks of gestation. Administration of Crinone was continued until a maximum of 12 weeks of gestation or until miscarriage or ectopic pregnancy was confirmed, whichever occurred first (Figure 1). The estrogen preparation was used until 8 or 9 weeks of gestation.

To eliminate as far as possible the influence of embryonic factors, FBT was performed only on morphologically good blastocysts with a Gardner's classification of 3BB or better.

### Study endpoints

The primary efficacy endpoint was the CPR per number of FBTs. Secondary efficacy endpoints included the biochemical pregnancy rate, early miscarriage rate, and ongoing pregnancy rate per FBT. Secondary safety endpoints were genital bleeding, abdominal pain, vaginal foreign substances, and genital itching; adverse events were graded on a scale of grade 1 (mildest) to 4 (most severe). The stratification factor was maternal age (i.e., younger than 38 years or 38 years and older).

### Study evaluations

Patients were assessed at the following times: 6 months prior to enrollment; on day −7 to −21, day 0 (day Crinone administration was started), day 4 to 5, and day 19 (±2 days); at 5 to 6 and 7 to 8 weeks of gestation; and, if applicable, after discontinuation and termination (up to 30 days after the last protocol treatment). Patient characteristics, levels of follicle‐stimulating hormone and other hormones, results of pregnancy tests, TVUS findings, concomitant medications, Crinone administration status, and safety evaluation were recorded.

### Statistical analysis

We analyzed the full analysis set (FAS; i.e., all eligible patients who received study treatment at least once and for whom some data were available), per‐protocol set (PPS; i.e., the population compliant with the study protocol), and safety analysis set (SAS; i.e., all patients who received study treatment at least once). The results of each analysis are presented for the whole group and by age group (<38 years and ≥38 years). Efficacy endpoints were evaluated in the FAS, and safety was assessed in the SAS. For continuous variables, we calculated the median (interquartile range), and for categorical variables, the proportions (%).

Confidence intervals for pregnancy rates (percentages) were estimated by the Wilson method. The statistical significance of differences in pregnancy rates between age groups was tested by Fisher's exact test.

As an ad hoc analysis, we compared the study results with the FET results in the ART2020 population from the JSOG ART2020 National Survey as a historical control. First, we drew a margins plot by using a logistic regression model, with clinical pregnancy as both a response and an indicator variable in the J‐FOCAL and ART2020 study groups, age categories (24 to 30 years, 31 to 37 years, and 38 to 42 years) as additional indicator variables, and the interaction of study group and age categories as explanatory variables. The statistical significance of the interaction terms was calculated with the likelihood ratio test. A logistic regression analysis stratified by age group was also performed, with clinical pregnancy as a response variable and study group as an explanatory variable.

Tests were two tailed, and an α of less than 0.05 was deemed statistically significant. All tabulations and statistical analyses were performed with Stata version 16.1 (Stata Corp., College Station, TX, USA).

## RESULTS

### Number of patients and enrollment period

**FIGURE 2:**
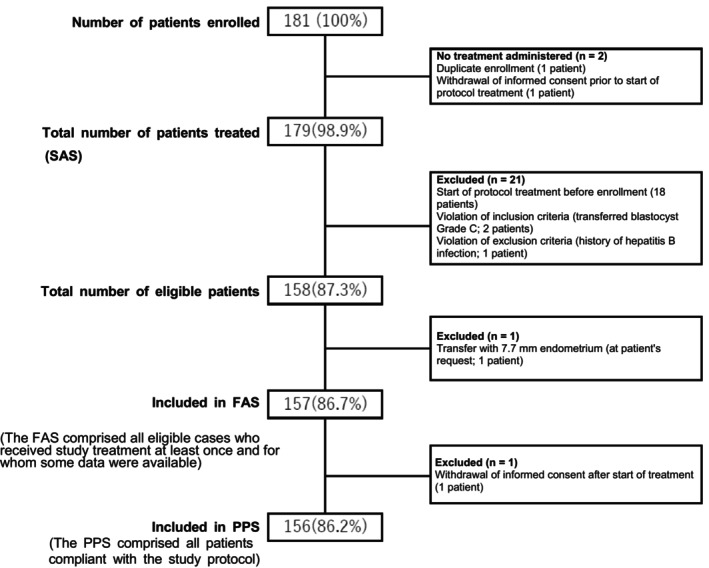
Flow chart describing the criteria for patient selection. FAS, full analysis set; PPS, per protocol set; SAS, safety analysis set.

The patient flow through the study and by analysis population are shown in Figure 2. Of the 181 patients enrolled, 179 exposed patients were included in the SAS (130 patients aged <38 years and 49 aged ≥38 years), 157 were included in the FAS (113 aged <38 years and 44 aged ≥38 years), and 156 were included in the PPS. The enrollment period was from May 17, 2018, to February 14, 2020. Because blastocyst transfer was not performed in one patient in the FAS, efficacy analyses were performed with the data from 156 patients.

### Patient characteristics

Patient characteristics and results of the pre‐enrollment hormone tests are shown in Table 2. Patient age ranged from 24 to 42 years, but the majority of patients (72.4%) were younger than 38 years.

**TABLE 2:** Demographic characteristics and hormone levels at baseline.

	Patients aged <38 years	Patients aged ≥38 years	Total group
	*n* = 113	*n* = 43	*N* = 156
Age, years	35.0 (32.0, 36.0) [24.0, 37.0]	40.0 (38.0, 41.0) [38.0, 42.0]	36.0 (33.0, 38.0) [24.0, 42.0]
Height, cm	160.0 (156.0, 163.0) [147.0, 172.0]	159.0 (156.0, 161.0) [150.0, 169.0]	160.0 (156.0, 163.0) [147.0, 172.0]
Weight, kg	52.5 (49.0, 58.0) [39.0, 84.0]	57.0 (49.0, 62.0) [42.4, 87.0]	53.0 (49.0, 60.0) [39.0, 87.0]
Body mass index, kg/m^2^	20.3 (19.1, 22.1) [16.2, 32.9]	22.0 (19.7, 24.2) [17.0, 36.2]	20.8 (19.3, 23.2) [16.2, 36.2]
Serum FSH level, mIU/mL	*N* = 110	*N* = 40	*N* = 150
	6.9 (5.4, 8.2) [0.1, 91.5]	7.3 (6.1, 9.0) [3.4, 18.6]	7.0 (5.6, 8.5) [0.1, 91.5]
Serum TSH level, mIU/mL	*N* = 99	*N* = 34	*N* = 133
	1.6 (1.2, 2.1) [0.1, 5.4]	1.6 (1.2, 2.0) [0.7, 2.9]	1.6 (1.2, 2.1) [0.1, 5.4]
Serum PRL level, ng/mL	*N* = 86	*N* = 29	*N* = 115
	13.8 (10.6, 18.2) [4.7, 33.2]	15.5 (11.7, 18.2) [8.4, 33.9]	14.2 (10.7, 18.2) [4.7, 33.9]

*Note*: Values are median (interquartile range) [minimum value, maximum value].

Abbreviations: FSH, follicle‐stimulating hormone; PRL, prolactin; TSH, thyroid‐stimulating hormone.

### Endometrial thickness, number of blastocysts transferred, and Gardner's classification of transferred blastocysts according to TVUS

TVUS measurements of endometrial thickness, number of blastocysts transferred, and Gardner's classification of the transferred blastocysts are shown in Table 3. The median endometrial thickness was 10.3 mm (min, 9.0; max, 11.4 mm; interquartile range, 7.5–15.0 mm), and only one patient had a thin uterine endometrium (<8 mm). All but one of the patients in the FAS underwent transfer with a single blastocyst.

**TABLE 3:** Endometrial thickness and information on blastocysts.

	Patients aged <38 years	Patients aged ≥38 years	Total
	*n* = 113	*n* = 43	*N* = 156
Endometrial thickness, median (IQR), [minimum, maximum], mm	10.6 (9.4, 11.5) [7.5, 15.0]	9.9 (8.4, 10.9) [8.0, 13.3]	10.3 (9.0, 11.5) [7.5, 15.0]
Number of blastocysts transferred, *n* (%)			
1	113 (100.0)	42 (97.7)	155 (99.4)
2	0 (0.0)	1 (2.3)	1 (0.6)
Gardner classification of transferred blastocyst, *n* (%)			
2	1 (0.9)	0 (0.0)	1 (0.6)
3	5 (4.4)	6 (14.0)	11 (7.1)
4	45 (39.8)	20 (46.5)	65 (41.7)
5	58 (51.3)	17 (39.5)	75 (48.1)
6	4 (3.5)	0 (0.0)	4 (2.6)
Gardner classification of transferred blastocyst/inner cell mass, *n* (%)			
A	73 (64.6)	21 (48.8)	94 (60.3)
B	40 (35.4)	22 (51.2)	62 (39.7)
Gardner classification of transferred blastocyst/trophic ectoderm, *n* (%)			
A	76 (67.3)	23 (53.5)	99 (63.5)
B	37 (32.7)	20 (46.5)	57 (36.5)

Abbreviation: IQR, interquartile range.

### Efficacy evaluation

**FIGURE 3:**
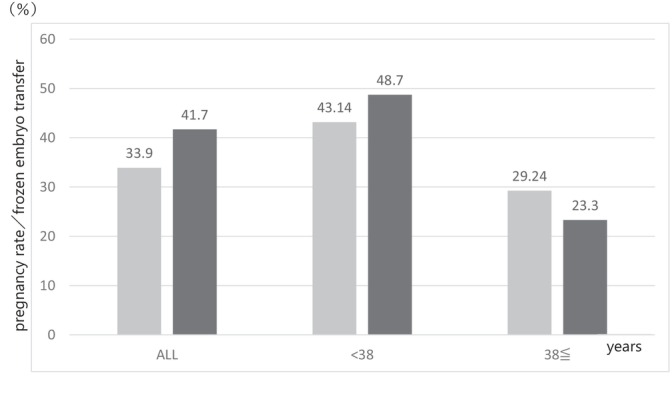
Comparison of clinical pregnancy rates between frozen embryo transfer in the Japan Society of Obstetrics and Gynecology ART2020 National Survey^3^ and frozen blastocyst transfer in the present study (J‐FOCAL). The gray bars indicate the clinical pregnancy rates in JSOG 2020, and the black bars indicate the clinical pregnancy rates in this study (J‐FOCAL). The rates in this study were higher than those reported in the JSOG ART2020 National Survey for the overall age group studied and for those under 38 years of age.

In the FAS, the CPR (95% confidence interval) was 41.7% (34.2–49.5) overall, 48.7% (39.7–57.8) in those aged under 38 years, and 23.3% (13.2–37.7) in those aged 38 and over; the respective biochemical pregnancy rates (95% confidence intervals) were 2.5% (1.0, 6.4), 2.7% (0.9, 7.5), and 2.3% (0.4, 12.1). The CPR per FBT was significantly higher in those aged under 38 years than in those aged 38 and over (*p* = 0.006), but the biochemical pregnancy rate per FBT was similar in both age groups (Table 4). The CPR in this study was higher than that reported in the JSOG ART2020 National Survey^3^ (Figure 3).

**TABLE 4:** Pregnancy rate per frozen blastocyst transfer.

A. Clinical pregnancy rate per frozen blastocyst transfer^a^			
	Age group		Total
	<38 years	≥38 years	
Number of FBTs	113	43	156
Clinical pregnancies	55	10	65
Clinical pregnancy rate, % (95% confidence interval)	48.7 (39.7, 57.8)	23.3 (13.2, 37.7)	41.7 (34.2, 49.5)

B. Biochemical pregnancy rate per frozen blastocyst transfer^b^			
	Age group		Total
	<38 years	≥38 years	
Number of FBTs	113	43	156
Number of confirmed miscarriages at 5–6 weeks' gestation	3	2	5
Number of positive quantitative tests for serum β‐hCG or hCG test on Day 19	3	1*	4*
Biochemical pregnancy rate, % (95% confidence interval)	2.7 (0.9, 7.5)	2.3 (0.4, 12.1)	2.5 (1.0, 6.4)

Abbreviations: FBT, frozen blastocyst transfer; hCG, human chorionic gonadotropin; TVUS, transvaginal ultrasound.

^a^ A clinical pregnancy was defined as a transvaginal ultrasound examination at 5 to 6 weeks' gestation that demonstrated a confirmed uterine or ectopic pregnancy.

^b^ A biochemical pregnancy was defined as a transvaginal ultrasound examination at 5 to 6 weeks' gestation that demonstrated the absence of a gestational sac and a confirmed miscarriage, accompanied by a positive serum beta human chorionic gonadotropin (β‐hCG) or hCG quantitative test on day 19 (±2).

**FIGURE 4:**
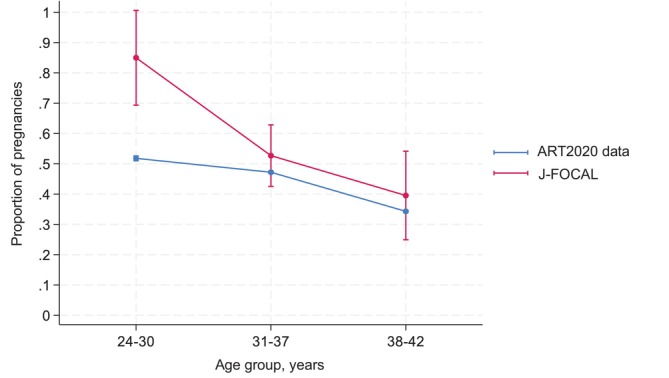
Predicted proportion of pregnancies. Error bars show 95% confidence intervals. ART2020 data (blue line) are from the Japan Society of Obstetrics and Gynecology ART2020 National Survey.^3^ A logistic regression model was performed, with clinical pregnancy as a response variable and study group (present study [J‐FOCAL] and ART2020 study), age group (24 to 30 years, 31 to 37 years, and 38 to 42 years), and interaction of study group and age group as explanatory variables. In the age group 24 to 30 years, the pregnancy rate was higher in the present study (J‐FOCAL) than in ART2020.

Figure 4 shows the margins plot comparing the results of the present study with the CPRs of FET from the JSOG ART2020 National Survey data by age group. In the group aged 24 to 30 years, the CPR was higher in the present study (J‐FOCAL) than in the JSOG ART2020 National Survey. The interaction between study group and age group was statistically significant (*p* = 0.04). The results of the age‐stratified logistic regression analysis are shown in Table 5. In the group aged 24 to 30 years, the odds ratio was significantly higher in the present study than in the JSOG ART2020 National Survey.

**TABLE 5:** Comparison of clinical pregnancy rates by age group between the present study (J‐FOCAL) and the Japan Society of Obstetrics and Gynecology ART2020 study.

Age category, years	Odds ratio (95% confidence interval)	*p*
24–30	5.27 (1.54, 18.00)	**0.008**
31–37	1.25 (0.83, 1.87)	0.29
38–42	1.25 (0.68, 2.31)	0.47

*Note*: Significant *p* values are written in bold. A logistic regression analysis stratified by age groups was performed with clinical pregnancy as the response variable and study group as the explanatory variable (present study [J‐FOCAL] and ART2020 study).

### Safety evaluation

Frequencies and severities of genital bleeding were as follows: day 0, 0.6% (grade 1); day 4 to 5, 0%; day 19, 5.8% (grade 1); 5 to 6 weeks' gestation, 8.2% (grade 1); 7 to 8 weeks' gestation, 7.0% (6.4%, grade 1; 0.6%, grade 2); and after discontinuation/termination, 7.0% (grade 1). Abdominal pain occurred in four patients (grade 1). No genital itching or vaginal foreign substances were observed. Other adverse events included threatened miscarriage in two patients (grade 1) and subchorionic hemorrhage in one patient (grade 1). No serious adverse events were observed.

## DISCUSSION

This multicenter study on the efficacy and safety of Crinone as the sole progesterone product for preparation of the endometrium during HRC‐FBT found a higher CPR in patients younger than 38 years old (48.7%) than in those aged 38 or older (23.3%). Furthermore, the total CPR (41.7%) was higher than that found with FET in the JSOG ART National Survey^3^ (36.0%; Figure 3). The maximum grade of genital bleeding was grade 2, and no serious adverse events occurred.

In addition to Crinone, three other transvaginal progesterone preparations and one intramuscular progesterone product are officially indicated for luteal support in Japan. The present study showed that the luteal support for FBT provided by Crinone, administered as the only progesterone preparation, was comparable to that of the treatments included in the JSOG ART2020 National Survey. A meta‐analysis also found no significant difference in the CPR or continued pregnancy rate between Crinone and other progesterone vaginal products.^10^ Additionally, Crinone was found to have advantages over other progesterone vaginal formulations in terms of luteal support in ART. For example, the analyses by Lan et al.^7^ and Ludwig et al.^10^ indicated that Crinone is associated with fewer adverse events and is more convenient than other progesterone vaginal formulations because it reduces vaginal discharge, causes less pain during administration, and is easier and quicker to administer. In addition, Simunic et al.^11^ found that Crinone was superior to other progesterone vaginal formulations because of the lower incidence of adverse events, simplicity of administration, and patient preference.

The present study found a statistically significant difference in both CPR and biochemical pregnancy rate per FBT between patients younger than 38 years and those aged 38 years or over (Table 5). The quality of transferred blastocysts is known to affect the pregnancy rate, so in this study, only blastocysts with a Gardner's score of 3BB or higher were transferred. The observed statistical difference in CPR between the two age groups is thought to be due to the quality of the transferred blastocyst in clinical pregnancies and the quality of the transferred blastocyst and age‐related factors in ongoing pregnancies.

To facilitate a comparison of the CPR after FBT in this study and after FET in the JSOG ART2020 National Survey, we performed an additional ad hoc analysis. Despite the limited number of cases and the wide confidence intervals, the results of this additional analysis indicated that (1) pregnancy rates decline with age; (2) for the entire target age group and for those under 38 years of age, the proportion of pregnancies from frozen blastocysts in this study was higher than that from frozen embryos in the national survey; and (3) the difference between the percentage of pregnancies from frozen blastocysts in this study and that observed from frozen embryos in the national survey was greater at younger ages because pregnancy rates were significantly higher in the present study, particularly among patients aged 30 years and younger. Because we were unable to perform propensity matching for age between patients in the present study and the JSOG ART2020 National Survey population, we performed an age‐group analysis (propensity matching was also not possible for transfer method, stage of transferred blastocysts/embryos, morphological classification of blastocysts/embryos, and number of transferred blastocysts/embryos). Therefore, the finding that the CPR in this study was higher than that in the ART2020 survey in the age‐group analysis should be interpreted with caution.

A limitation of the present study is that it did not have a control arm and was compared with a National Survey. FBT was used in the present study, whereas FET was used in the National Survey because it cannot distinguish between the two stages of embryo transfer.

In conclusion, this study demonstrated the efficacy and safety of Crinone as the sole progesterone vaginal application for luteal support during HRC‐FBT and showed that the CPR was not lower than that for FET in the JSOG ART2020 National Survey.

## AUTHOR CONTRIBUTIONS

**Akiko Takashima:** Data curation; investigation; resources; writing – original draft. **Yukihiro Shibui:** Investigation. **Toshio Hamatani:** Investigation. **Akihiko Suenaga:** Investigation. **Yoshimitsu Kuwabara:** Investigation. **Fuminori Kimura:** Investigation. **Koji Nakagawa:** Investigation; writing – review and editing. **Yusuke Fukuda:** Conceptualization; investigation; methodology; visualization. **Shoji Tokunaga:** Data curation; formal analysis; software. **Yukiko Katagiri:** Conceptualization; project administration; supervision; writing – review and editing.

## CONFLICT OF INTEREST STATEMENT

The authors declare no conflict of interest. Dr. Kuwabara, Yoshimitsu and Dr. Kimura, Fuminori are the Editorial Board members of JOGR Journal and a co‐authors of this article. To minimize bias, they were excluded from all editorial decision‐making related to the acceptance of this article for publication.

## Data Availability

The data that support the findings of this study are available on request from the corresponding author. The data are not publicly available due to privacy or ethical restriction. Only anonymized and minimal data will be disclosed in the institutional repository. https://toho.repo.nii.ac.jp/records/2015701.

## APPENDIX 1

**Inclusion criteria**

1 Patients of Japanese ethnicity
2 Patients undergoing frozen blastocyst transfer (FBT) and scheduled to use Crinone for luteal support during a hormone replacement cycle
3 Patients with transferable frozen blastocysts (no blastocysts of Gardner's grade C) who are eligible for hormone replacement cycle FBT
4 Healthy premenopausal women aged 42 years or younger at the time of blastocyst transfer who wish to conceive a child
5 Patients must have a normal cervical cytology result, which is defined as a negative result for intraepithelial lesion or malignancy, atypical squamous cells of undetermined significance, and human papillomavirus (HPV), and must have had this result within 12 months prior to the date of informed consent. The results of the tests may be declared by the patient themselves (a record of the results should be kept in the medical record). In the event that cytology has not been performed during the aforementioned period, cervical cytology and, if necessary, HPV testing should be conducted during the screening period.
6 Patients who have provided their written informed consent to participate in the study
7 Patients participating in this study for the first time

**Exclusion criteria**

1 Patients with the potential for spontaneous ovulation (presence of mature follicles)
2 Patients with a history of repeated miscarriages (defined as three or more previous spontaneous miscarriages)
3 Patients with three or more consecutive cycles of discontinuation or failure (no clinical pregnancy) in the past
4 Patients who received ovarian stimulation with clomiphene citrate in the previous cycle and have not menstruated since egg retrieval
5 Patients with undiagnosed irregular bleeding
6 Patients with contraindications to pregnancy
7 Patients with endometriosis, uterine fibroids, uterine adenomyosis, or endometrial polyps requiring treatment
8 Patients with an ectopic pregnancy within 3 months prior to the date of informed consent
9 Patients with unexplained ovarian enlargement or cysts
10 Patients who are breastfeeding
11 Patients who are positive for human immunodeficiency virus, have a history of hepatitis B or C virus infection, or have been diagnosed with active hepatitis B or C virus infection
12 Patients with a history of allergy or hypersensitivity to progesterone‐containing preparations and/or their additives or in whom progesterone preparations are contraindicated
13 Patients with a history of alcohol or drug abuse or suspected alcohol or drug abuse within 5 years prior to the date of informed consent
14 Patients with active thrombophlebitis, thromboembolic disease or stroke, or a history of these conditions
15 Patients deemed unsuitable for participation in the study by the principal site investigator or investigator

## APPENDIX 2

**Protocol treatment discontinuation and completion criteria**

**Definition of completion of protocol treatment**

Pregnancy is confirmed by the presence of a fetal heartbeat at 7 to 8 weeks' gestation; however, Crinone may be administered up to 12 weeks' gestation at the discretion of the investigator.

**Criteria for discontinuation of protocol treatment**

Protocol treatment is discontinued if any of the following occur:

1 A negative serum beta human chorionic gonadotropin or human chorionic gonadotropin quantification test result on Day 19 (± 2 days)
2 If miscarriage or ectopic pregnancy is confirmed
3 If other progesterone preparations are required
4 If serious adverse events, such as anaphylactic reactions, occur and it is deemed difficult to continue the study
5 If it is deemed difficult to continue because of the development of a new disease or exacerbation of a complication
6 In other cases where the investigator deems it difficult to continue the study
7 If the study patient requests the discontinuation of treatment
8 In the case of unfeasible follow‐up
